# Challenges to human papillomavirus vaccination among young girls in Morocco: A national cross-sectional study

**DOI:** 10.1371/journal.pone.0347783

**Published:** 2026-05-20

**Authors:** Touria Essayagh, Meriem Essayagh, Housnia Slibani, Soukayna El Hachimi, Kaoutar Nmila, Khaoula Addakiri, Rehmatullah Zadran, Sanah Essayagh

**Affiliations:** 1 Hassan First University of Settat, Institut Supérieur des Sciences de la Santé, Laboratoire des Sciences et Ingénierie Biomédicales, Biophysique et Santé, Settat, Morocco; 2 Office National de Sécurité Sanitaire des produits Alimentaires, Meknès, Morocco; 3 National Infectious Diseases Hospital Laboratory, Kabul, Afghanistan; 4 Hassan First University of Settat, Faculté des Sciences et Techniques, Laboratoire Agroalimentaire et Santé, Settat, Morocco; Federal University Otuoke, NIGERIA

## Abstract

**Background:**

The human papillomavirus (HPV) vaccine helps significantly reduce the incidence of cervical cancer. However, in many countries, its adoption remains limited. In this context, our study aimed to measure the prevalence of HPV vaccination hesitancy among young girls and to identify its associated risk factors.

**Methods:**

From 10^th^ April to 30^th^ June 2025, a cross-sectional survey was conducted in Morocco among girls aged 11–14 attending middle school. Face-to-face interviews gathered sociodemographic data, HPV knowledge, and vaccination attitudes. Multivariate logistic regression identified risk variables for vaccine hesitancy.

**Results:**

A total of 1,836 participants were collected with a mean age of 13.0 ± 0.8 years. The percentage of vaccine hesitancy reached 88.8%. The main risk factors were unvaccinated pairs (AOR = 11.78; CI [7.67–18.0]), paternal illiteracy (AOR = 7.67; [1.99–29.55]), low recommendation of the vaccine by health personnel (AOR = 4.14; [2.44–7.02]), belief that the vaccine promotes sexual activity (AOR = 3.10; [2.01–4.79]), insufficient knowledge of the target population (AOR = 2.51; [1.63–3.85]), perception of adverse effects (AOR = 1.97; [1.31–2.98]), insufficient knowledge of the number of doses required (AOR = 1.76; [1.06–2.92]), fear of vaccination (AOR = 1.71; [1.14–2.58]), fear of regretting vaccination (AOR = 1.55; [1.02–2.36]), and age (AOR = 1.34; [1.06–1.69]).

**Conclusion:**

HPV vaccination hesitancy remains high and is shaped by social, educational, and cultural factors. These findings underscore the need for targeted educational initiatives and increased awareness among healthcare professionals. Integrating vaccination into school health programs may help improve uptake.

## Introduction

Human papillomavirus (HPV) is a prevalent sexually transmitted infection and a leading cause of cervical cancer worldwide [1]. Globally, cervical cancer was the fourth most common cancer among women in 2022, with approximately 662,301 new cases and 348,874 deaths reported annually [2]. HPV infection is typically asymptomatic, and in about 90% of cases, the immune system clears the virus within one to two years without long-term consequences [1,3]. However, persistent infection can lead to genital warts and, over 10–20 years, precancerous lesions of the cervix that may progress to invasive cancer [1]. Over 200 HPV genotypes have been identified and classified according to their oncogenic risk, with genotypes 16 and 18 alone accounting for around 70% of cervical cancer cases globally [4,5]. In the Eastern Mediterranean region, a 2019 meta-analysis including 5,990 women with pre-cervical cancer and cervical cancer showed that HPV genotypes 16 and 18 were responsible for 74.5% of invasive cases [6].

In Morocco, cervical cancer remains a significant health burden. In 2024, 2,644 new cases and 1,468 deaths were reported [2,7]. The disease negatively impacts women’s quality of life, causing persistent pain, abnormal vaginal discharge, chronic bleeding, and fistulas, which may result in social isolation and psychological distress [8]. Risk factors such as low parental literacy and limited access to healthcare services are particularly relevant in understanding barriers to prevention in Moroccan communities.

Prophylactic HPV vaccination is an effective strategy to prevent infection and subsequent cervical cancer [9]. Vaccination of girls aged 9–14 before sexual debut is highly recommended [3]. The World Health Organization’s global strategy to eliminate cervical cancer emphasizes achieving 90% HPV vaccination coverage among girls by age 15 [3]. In Morocco, HPV vaccines (Cervarix and Gardasil) were introduced in 2008 for girls aged 11–25, ideally before sexual debut, with a dose priced at US$41.5—substantially higher than in socioeconomically comparable countries, where the cost is US$9.34 [2]. Since 2022, HPV vaccination has been included in the national immunization program and offered free of charge, yet coverage remains below 21% nationally [10].

Despite vaccine availability, hesitancy remains a critical challenge. The World Health Organization defines vaccine hesitancy as a delay in acceptance or refusal of vaccination despite the availability of services [11,12]. Contributing factors include limited knowledge of HPV and cervical cancer, doubts about vaccine effectiveness, low perception of personal risk, financial constraints, and difficulties accessing healthcare [13,14]. In Morocco, however, no studies have specifically examined determinants of HPV vaccine hesitancy among girls targeted by the national program, although 2024 data in Morocco report a literacy rate of 92,1% amongst girls aged 11–14 [15]. To address this gap, our study aimed to measure the prevalence of HPV vaccine hesitancy and identify associated risk factors, thereby contributing to strategies to improve sexual and reproductive health and reduce premature mortality.

## Methods

### Study design and population

Between 10^th^ April and 30^th^ June 2025, a cross-sectional survey was conducted among girls aged 11–14 in middle schools in the provinces of Settat, Khouribga, Kenitra, and Tinghir. The sampling strategy used a two-stage stratified survey design, with middle schools serving as the primary unit and classes serving as the secondary unit. A comprehensive and numbered list of all middle schools in the provinces was obtained from the National Education Delegation of Settat, Khouribga, Kenitra, and Tinghir. A random selection process was applied to determine the primary unit (middle schools). Once the primary units were identified, a list of all classes containing girls aged 11–14 was obtained from the middle school administration. Each class was then selected by simple random sampling. Parents or legal guardians of all girls aged between 11 and 14 years in each selected class were informed about the study objectives through an information letter distributed in sealed envelopes via the students by investigators. Written informed consent was obtained from parents or guardians and returned to the investigators through the school administration.

Only participants whose parents or guardians provided written consent and whom themselves gave informed assent were included in the study. Prior to data collection, all participating girls completed a written assent form, confirming their voluntary participation. Individual interviews were conducted with the girls to complete the questionnaire. Girls aged 11–14 years who had ever engaged in sexual activity were excluded from the study. In addition, girls with medical contraindications to HPV vaccination were also excluded from participation.

### Sample size determination

Assuming an expected HPV vaccine hesitancy percentage of 50%, a 5% margin of error, and a 19.5% non-response rate, the required sample size was calculated to be 459 participants per province. These calculations were performed using Epi Info 7 software.

### Data collection

A standardized questionnaire collected sociodemographic data, assessed knowledge of HPV infection and cervical cancer, and explored perceptions of HPV severity, vulnerability, benefits, risks, and barriers to HPV vaccination was administered during face-to-face interviews between trained interviewers and girls aged 11–14 years.

### Operational definitions

Vaccination hesitancy was assessed through a series of questions. Participants were first asked about their HPV vaccination status (“Are you vaccinated against HPV?”). If they answered no, two follow-up questions assessed whether non-vaccination was due to delayed acceptance or outright refusal. Hesitancy was considered present if either response was affirmative.

Perceived susceptibility to HPV was measured by participants’ self-reported likelihood of infection, while beliefs about severity reflected their evaluation of the health threat. Perceived benefits were assessed through confidence in the vaccine’s protective ability, and perceived risks were captured by concerns about safety and potential side effects.

Perceived barriers included fears that vaccination might encourage sexual activity. Social influence was measured by participants’ perceptions of how family and peers affected their vaccination decisions. Fear of regret was assessed using three yes/no items: fear of being vaccinated, fear of regretting vaccination, and fear of regretting not being vaccinated.

Finally, prior vaccination behavior was evaluated with three yes/no questions: whether participants had followed the recommended vaccine schedule, completed all required vaccinations, and ever refused a recommended vaccine.

Relationship with the healthcare system was assessed by questions about the distance, travel time, and transportation mode to the nearest healthcare center. Unsatisfactory relationship with the healthcare system was defined as living more than six kilometers from a health facility, requiring over 30 minutes of travel, or relying on public transportation [16–19].

### Data management and analysis

Data analysis was performed using Epi Info version 7.2.0.1. A significance level of 0.05 was applied to all statistical tests, and all tests were two-tailed. Results are presented as mean ± standard deviation for quantitative variables and as percentages for qualitative variables. To address the primary objective of the study, a univariate analysis was first conducted to identify factors associated with vaccine hesitancy, which were subsequently included in the multivariate analysis. Descriptive analysis was initially performed for the entire study population, followed by subgroup analyses. The relevance of qualitative variables was assessed using the Pearson chi-square test, while the Student’s t-test was applied to quantitative variables. The association between each potential risk factor and vaccine hesitancy was determined using the odds ratio (OR) and its 95% confidence interval (CI).

### Ethical considerations

Ethical approval for this study was obtained from the Ethics Committee of Mohammed V University in Rabat, Morocco (reference no. 65/25). The study was conducted in accordance with the ethical principles set forth in the Declaration of Helsinki. All participants and their parents were informed of the study’s objective and data confidentiality, and both parents and girls provided written consent before participating. Participation was voluntary, and participants could withdraw at any time without consequence.

## Results

**1. Socioeconomic and demographic characteristics**

Table 1 summarizes the socioeconomic and demographic characteristics of girls aged 11–14 years. Of the 1,836 participants recruited, 1,631 (88.8%) were hesitant about vaccination. The mean age of the participants was 13.0 ± 0.8 years; 1,062 (57.8%) lived in urban areas, and 1,675 (91.2%) had married parents. Furthermore, 1,569 (85.5%) reported a monthly family income of less than $400, 523 (28.5%) had mothers who could not read or write, and 912 (49.7%) reported being unsatisfied with the health system (Table 1).

**Table 1:** The socioeconomic and demographic characteristics of the participants, Morocco, 2025.

Quantitative variables	Total	HPV vaccination hesitancy	HPV vaccination non hesitancy	*P-value*
Participants’ total no. (%)	1,836 (100.0)	1,631 (88.8)	205 (11.2)	
Age in years ± sd	13.0 ± 0.8	13.0 ± 0.8	12.9 ± 0.9	0.10
**Qualitative variables no. (%)**				
Area of residence				0.76
Rural	774 (42.2)	690 (42.3)	84 (41.0)	
Urban	1,062 (57.8)	941 (57.7)	121 (59.0)	
Marital status of parents				0.43
Uncoupled^*†*^	161 (8.8)	140 (08.6)	21 (10.2)	
Coupled^*^	1,675 (91.2)	1,491 (91.4)	184 (89.8)	
Girl’s level of education				0.98
First year of middle school	945 (51.4)	839 (51.4)	106 (51.7)	
Second year of middle school	693 (37.8)	615 (37.7)	78 (38.1)	
Third year of middle school	198 (10.8)	177 (10.9)	21 (10.2)	
Mother’s level of education				0.93
Cannot read or write	523 (28.5)	464 (28.5)	59 (28.8)	
Can read and write	1,313 (71.5)	1,167 (71.5)	146 (71.2)	
Father’s level of education				<0.001
Cannot read or write	155 (08.4)	152 (09.3)	3 (01.5)	
Can read and write	1,681 (91.6)	1,479 (90.7)	202 (98.5)	
Monthly income per household ($)				0.07
<400	1,569 (85.5)	1,385 (84.9)	184 (89.8)	
≥400	267 (14.5)	246 (15.1)	21 (10.2)	
Relationship with the healthcare system				0.20
Unsatisfactory	912 (49.7)	819 (50.2)	93 (45.4)	
Satisfactory	924 (50.3)	812 (49.8)	112 (54.6)	

HPV stands for human papillomavirus.

*: Coupled refers to being married or in a concubine relationship.

†: Uncoupled means single, divorced, or widowed.

When the conditions were valid, the Pearson chi-2 test estimated the association between the dependent variable and the independent variables. We used a comparison test of two means for the quantitative variables; a p-value less than 0.05 was considered significant.

**2. General knowledge about human papillomavirus, cervical cancer, and the vaccine**

Table 2 presents participants’ level of knowledge regarding HPV, cervical cancer, and the HPV vaccine. A total of 1,410 (76.8%) participants had never heard of HPV, while 959 (52.2%) said they did not know about cervical cancer. Furthermore, 943 (51.4%) did not know who the HPV vaccination was intended for, and 1,599 (87.1%) did not know how many doses should be given. A total of 1,228 (66.9%) believed that the level of information supplied by the media about the HPV vaccine was unsatisfactory.

**Table 2:** General knowledge about human papillomavirus infection, cervical cancer, and the vaccine, girls, Morocco, 2025.

Qualitative variables	Total (n = 1,836)	HPV vaccination hesitancy (n = 1,631)	HPV vaccination non hesitancy (n = 205)	*P-value*
Having already heard of HPV infection				<0.001
No	1,410 (76.8)	1,279 (78.4)	131 (63.9)	
Yes	426 (23.2)	352 (21.6)	74 (36.1)	
General knowledge about HPV infection				0.59
Unsatisfactory	1,169 (63.7)	1,042 (63.9)	127 (61.9)	
Satisfactory	667 (36.3)	589 (36.1)	78 (38.1)	
Having been in contact with a person infected with HPV				0.24
No	1,765 (96.1)	1,571 (96.3)	194 (94.6)	
Yes	71 (03.9)	60 (03.7)	11 (05.4)	
Having already heard of cervical cancer				<0.001
No	959 (52.2)	885 (54.3)	74 (36.1)	
Yes	877 (47.8)	746 (45.7)	131 (63.9)	
General knowledge about cervical cancer				0.87
No	1,205 (65.6)	1,069 (65.5)	136 (66.3)	
Yes	631 (34.4)	562 (34.5)	69 (33.7)	
Knowledge of the population targeted by the HPV vaccine				<0.001
No	943 (51.4)	903 (55.4)	40 (19.5)	
Yes	893 (48.6)	728 (44.6)	165 (80.5)	
Knowledge of the number of doses to administer regarding the HPV vaccine				<0.001
No	1,599 (87.1)	1,441 (88.3)	158 (77.1)	
Yes	237 (12.9)	190 (11.7)	47 (22.9)	
Perception of the level of information provided by the media on the HPV vaccine				0.20
Unsatisfactory	1,228 (66.9)	1,099 (67.4)	129 (62.9)	
Satisfactory	608 (33.1)	532 (32.6)	76 (37.1)	

HPV stands *for* human papillomavirus.

When the conditions were valid, the Pearson chi-2 test estimated the association between the dependent variable and the independent variables; a p-value less than 0.05 was considered significant.

**3. Perceptions and concerns regarding the human papillomavirus vaccine**

Among the 1,836 participants, 727 (39.6%) perceived the HPV vaccine as risky with potential adverse effects, 869 (47.3%) believed it could encourage sexual activity, 1,224 (66.7%) reported fear of being vaccinated, and 1,120 (61%) expressed concern that they might regret getting vaccinated “Table 3”.

**Table 3:** Perceptions and concerns regarding the human papillomavirus vaccine, Morocco, 2025.

Qualitative variables	Total (n = 1,836)	HPV vaccination hesitancy (n = 1,631)	HPV vaccination non hesitancy (n = 205)	*P-value*
Susceptibility to HPV infection				0.22
No	720 (39.2)	648 (39.7)	72 (35.1)	
Yes	1,116 (60.8)	983 (60.3)	133 (64.9)	
Perceived beliefs about the seriousness of HPV infection				0.69
No	325 (17.7)	291 (17.8)	34 (16.6)	
Yes	1,511 (82.3)	1,340 (82.2)	171 (83.4)	
Perceived beliefs about HPV vaccine benefits				0.32
No	403 (21.9)	364 (22.3)	39 (19.0)	
Yes	1,433 (78.1)	1,267 (77.7)	166 (8.0)	
HPV vaccine adverse reaction				0.02
No	727 (39.6)	661 (40.5)	66 (32.2)	
Yes	1,109 (60.4)	970 (59.5)	139 (67.8)	
HPV vaccine would encourage sexual activity				<0.001
Yes	869 (47.3)	817 (50.1)	52 (25.4)	
No	967 (52.7)	814 (49.1)	153 (74.6)	
**Fear of regret over HPV vaccination**				
Fear of having vaccinated with the anti-HPV vaccine				<0.001
Yes	1,224 (66.7)	1,123 (68.9)	101 (49.3)	
No	612 (33.3)	508 (31.1)	104 (50.7)	
Fear of regretting having vaccinated with the anti-HPV vaccine				0.002
Yes	1,120 (61.0)	1,015 (62.2)	105 (51.2)	
No	716 (39.0)	616 (37.8)	100 (48.8)	
Fear of regretting not having been vaccinated with the anti-HPV vaccine				0.40
Yes	1,084 (59.0)	957 (58.7)	127 (61.9)	
No	752 (41.0)	674 (41.3)	78 (38.1)	

HPV stands *for* human papillomavirus.

When the conditions were valid, the Pearson chi-2 test estimated the association between the dependent variable and the independent variables; a p-value less than 0.05 was considered significant.

**4. Environnemental influences**

Table 4 presents the impact of environmental determinants on HPV vaccination among young girls. Among the participants, 642 (35.0%) reported that their social circle had a negative influence on their decision to get vaccinated. In addition, 1,612 (87.8%) indicated that no girl their age in their entourage had received the vaccine. Regarding information from health professionals, 1,596 participants (86.9%) reported that they had not been informed about the availability of the vaccine at health centers, 1,498 (81.6%) were unaware that it was offered free of charge, and 1,556 (84.7%) indicated that health professionals had not recommended to their parents that they get vaccinated.

**Table 4:** Influences of environmental determinants on the decision to vaccinate with the HPV vaccine, Morocco, 2025.

Qualitative variables	Total (n = 1,836)	HPV vaccination hesitancy (n = 1,631)	HPV vaccination non hesitancy (n = 205)	*P-value*
Media influence on HPV vaccination				0.22
Negative	720 (39.2)	648 (39.7)	72 (35.1)	
Positive	1,116 (60.8)	983 (603)	133 (64.9)	
Influence of societal norms on HPV vaccination				0.02
Negative	642 (35.0)	585 (35.9)	57 (27.8)	
Positive	1,194 (65.0)	1,046 (64.1)	148 (72.2)	
**Influence of previous experiences with cancer**				
Have already been in contact with a person suffering from cancer				0.46
No	1,561 (85.0)	1,390 (85.2)	171 (83.4)	
Yes	275 (15.0)	241 (14.8)	34 (16.6)	
Have already had cancer				1.0
No	1,778 (96.6)	1,579 (96.8)	199 (97.1)	
Yes	58 (03.2)	52 (03.2)	6 (02.9)	
**Previous vaccination behavior**				
Have already be vaccinated previously according to the vaccination schedule				0.80
No	194 (10.6)	174 (10.7)	20 (09.8)	
Yes	1,642 (89.4)	1,457 (89.3)	185 (90.2)	
Have complied in the past with all the vaccines recommended according to the vaccination schedule				0.72
Yes	209 (11.4)	184 (11.3)	25 (12.2)	
No	1627 (88.6)	1,447 (88.7)	180 (87.8)	
Have refused to comply with all vaccinations recommended for their children in the past				0.37
Yes	174 (09.5)	151 (09.3)	23 (11.2)	
No	1662 (90.5)	1,480 (90.7)	182 (88.8)	
The health worker informs the parents that the health center has a vaccine against HPV				<0.001
No	1596 (86.9)	1,468 (90.0)	128 (62.4)	
Yes	240 (13.1)	163 (10.0)	77 (37.6)	
The health worker informs the parents about the free HPV vaccine at the health center				<0.001
No	1498 (81.6)	1,394 (85.5)	104 (50.7)	
Yes	338 (18.4)	237 (14.5)	101 (49.3)	
The health worker advises parents to vaccinate their daughters against HPV at the health center				<0.001
No	1556 (84.7)	1,454 (89.2)	102 (49.8)	
Yes	280 (15.3)	177 (10.8)	103 (50.2)	
Peers already vaccinated				<0.001
No	1612 (87.8)	1,511 (92.6)	101 (49.3)	
Yes	224 (12.2)	120 (07.4)	104 (50.7)	

HPV stands for human papillomavirus.

**5. Bivariate analysis**

HPV vaccination hesitancy affected 1631 (88.8%) participants. Following the bivariate analysis, the p-value cutoff was set at p ≤ 0.05. According to the bivariate analysis, we identified 14 risk factors associated with vaccination hesitancy: 1. a father who cannot read or write (p < 0.001); 2. not having already heard of HPV infection (p < 0.001); 3. not having ever heard of cervical cancer (p < 0.001); 4. lack of knowledge of the population targeted by the HPV vaccine (p < 0.001); 5. lack of knowledge of the number of doses of HPV vaccine to administer (p < 0.001); 6. belief that the vaccine has adverse effects (p = 0.02); 7. belief that the vaccine encourages sexual activity (p < 0.001); 8. fear of being vaccinated (p < 0.001); 9. fear of regretting having been vaccinated (p = 0.002); 10. influence of societal norms (p = 0.02); 11. insufficient awareness of health personnel regarding the vaccine (p < 0.001); 12. insufficient awareness of health personnel about the free vaccine (p < 0.001); 13. insufficient recommendation of the vaccine by health personnel (p < 0.001); 14. peers not already vaccinated with the HPV vaccine (p < 0.001) “Table 5”.

**Table 5:** Multivariate analysis (Odds ratio, P-value) of human papillomavirus vaccination hesitancy risk factors among young girls, Morocco, 2025.

	Bivariate analysis for human papillomavirus vaccination hesitancy		Multivariate analysis complete model for human papillomavirus vaccination hesitancy	
**Variables**	**AOR [95%CI]**	***P-value***	**AOR [95%CI]**	***P-value***
Age in years	1.15 [0.96-1.38]	0.10	1.34 [1.06-1.69]	0.01
Father’s level of education: Cannot read or write	6.91 [2.18-21.9]	<0.001	7.67 [1.99-29.55]	0.003
Not having heard of HPV infection	2.05 [1.50-2.79]	<0.001	1.01 [0.63-1.60]	0.96
Not having heard of cervical cancer	2.1 [1.55-2.83]	<0.001	1.19 [0.78-1.81]	0.41
Lack of knowledge of the population targeted by the HPV vaccine	5.11 [3.57-7.32]	<0.001	2.51 [1.63_3.85]	<0.001
Not knowing the number of doses to administer	2.25 [1.57-3.23]	<0.001	1.76 [1.06-2.92]	0.02
Belief that the vaccine has adverse effects	1.43 [1.05-1.95]	0.02	1.97 [1.31-2.98]	0.001
Belief that the vaccine encourages sexual activity in young girls	2.95 [2.12-4.10]	<0.001	3.10 [2.01-4.79]	<0.001
Fear of getting vaccinated	2.27 [1.69-3.95]	<0.001	1.71 [1.14-2.58]	0.009
Fear of regretting having been vaccinated	1.56 [1.17-2.10]	0.002	1.55 [1.02-2.36]	0.03
Influence of societal norms	1.45 [1.05-2.00]	0.02	1.09 [0.73-1.65]	0.64
Insufficient awareness of the vaccine among healthcare staff	5.41 [3.91-7.50]	<0.001	1.41 [0.79-2.50]	0.23
Insufficient awareness among healthcare staff regarding the free nature of the vaccine	5.71 [4.20-7.76]	<0.001	1.28 [0.77-2.11]	0.33
Insufficient recommendation regarding the use of vaccines by healthcare personnel	8.29 [6.05-11.37]	<0.001	4.14 [2.44-7.02]	<0.001
Close contact not already vaccinated against HPV	12.96 [9.31-18.95]	<0.001	11.78 [7.67-18.0]	<0.001

HPV stands for human papillomavirus; AOR: Adjusted Odds Ratio, CI: Confidence Interval.

**6. Multivariate analysis**

**Fig 1:**
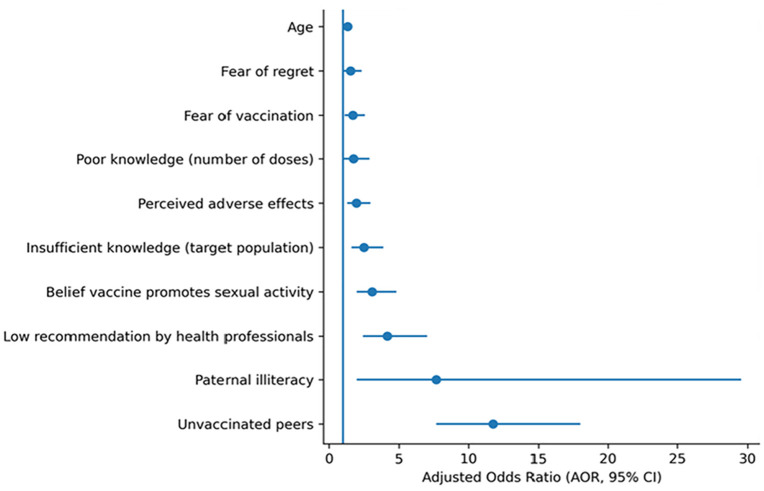
Determinants of human papillomavirus vaccine hesitancy (multivariate analysis; adjusted odds rations with 95% confidence intervals). AOR: Adjusted Odds Ratio, CI: Confidence Interval.

After controlling for other variables, ten factors were found to be significantly associated with HPV vaccination hesitancy. The strongest association was observed for peers not previously vaccinated against HPV (AOR 11.78; 95% CI [7.67–18.0]), followed by having a father who is illiterate (AOR 7.67; 95% CI [1.99–29.55]) and insufficient recommendation of the vaccine by health personnel (AOR 4.14; 95% CI [2.44–7.02]). Other significant factors included the belief that the vaccine encourages sexual activity (AOR 3.10; 95% CI [2.01–4.79]), lack of knowledge about the population targeted by the vaccine (AOR 2.51; 95% CI [1.63–3.85]), and concerns about potential adverse effects of the vaccine (AOR 1.97; 95% CI [1.31–2.98]). Additional factors were lack of knowledge regarding the required number of doses (AOR 1.76; 95% CI [1.06–2.92]), fear of being vaccinated (AOR 1.71; 95% CI [1.14–2.58]), fear of regretting vaccination (AOR 1.55; 95% CI [1.02–2.36]), and age (AOR 1.34; 95% CI [1.06–1.69]) “Table 5” and “Fig 1”.

## Discussion

HPV vaccination is a major strategic focus in the fight against cervical cancer. However, vaccine hesitancy has hampered its rollout over the past decade [20], leading to negative repercussions for women’s sexual and reproductive health. In our study, the hesitancy rate reached 88.8%. By comparison, a survey conducted in China in 2022 with 728 participants reported a rate of 62.4% [21] while in Ethiopia, in the same year, a study including 423 participants reported a rate of 27.9% [22].

The presence of an unvaccinated peer of the same age in one’s social circle is significantly associated with vaccine hesitancy [23]. This influence is explained by the fact that peer behavior can shape risk perception and affect vaccine acceptance by an individual or their parents [24]. Exposure to this type of social model can thus reduce confidence in vaccination, just as the absence of advice or family support can limit acceptance. Peer-led education initiatives have been shown to significantly enhance vaccination knowledge and intentions, particularly when combined with health expert involvement, highlighting the value of using peer ambassadors in HPV promotion campaigns [25].

Our results indicate that paternal illiteracy is a risk factor for HPV vaccination hesitancy. These findings align with previous studies demonstrating that higher health literacy is strongly associated with increased vaccine acceptance [26,27]. Indeed, individuals with limited reading and writing skills have greater difficulty accessing reliable information about HPV and its risks, which can increase distrust of healthcare professionals’ recommendations and promote the spread of rumors or misinformation [28].

To overcome this obstacle, tailored educational and communication strategies—such as simplified narrative videos, illustrated fact sheets, and culturally relevant messages—have proven effective in improving HPV vaccination understanding and coverage in low-literacy communities [29].

In our survey, insufficient HPV vaccine recommendations from healthcare professionals emerged as a risk factor for vaccination hesitancy. When a professional is unclear or expresses doubts about the vaccine’s usefulness or safety, that can lead to mistrust among patients or their parents. Conversely, clear, confident, and transparent communication that reassures families about vaccine safety and emphasizes its role in cancer prevention fosters greater trust, leading to better acceptance and higher vaccination prevalence [30,31]. A meta-analysis of 59 studies mentioned that receiving a recommendation from a professional is associated with a very significant increase in HPV vaccination initiation [32]. Research demonstrates that high-quality vaccine recommendations, particularly those emphasizing same-day vaccination, significantly increase the likelihood of initiating and completing the HPV vaccination schedule [33]. Training healthcare providers in effective communication significantly increases HPV vaccine uptake by improving recommendation practices, including the use of prompts, and feedback [34].

In our study, the belief that the HPV vaccine encourages early or risky sexual activity among young people was associated with vaccine hesitancy. However, several epidemiological studies have refuted this idea. A longitudinal study conducted in the United States on 1,398 adolescent girls between 2006 and 2010 found no association between HPV vaccination and earlier sexual initiation or an increase in risky sexual behaviors [35]. Similarly, a Canadian survey of 260 young women showed that vaccination did not change sexual behaviors but helped strengthen their sense of protection and confidence in preventing sexually transmitted infections [36]. These results are confirmed by a more recent study conducted in the United Kingdom between 2010 and 2016 with 4482 adolescent girls, which demonstrated that HPV vaccination was not correlated with accumulated sexual activity or a decrease in the use of protection methods [37].

A lack of knowledge about the target population for the HPV vaccine is a major barrier to its acceptance and contributes to vaccine hesitancy. Many parents and adolescents are unaware that the vaccine should be administered before the onset of sexual activity, generally between the ages of 9 and 14. A study conducted in Spain among 1,405 parents showed that limited knowledge of the recommended vaccination age significantly increased vaccine hesitancy [38]. Similarly, a 2023 meta-analysis in Ethiopia confirmed that poor awareness of the target population represents one of the main barriers to vaccination coverage [39]. These findings are consistent with those of an international systematic review and meta-analysis, which highlight that, in various contexts, a lack of knowledge about the recommended age group remains a determining factor in vaccine hesitancy [40]. Educational interventions delivered directly to adolescents—especially when theory‑based and tailored to the audience—have shown improvements in HPV knowledge, attitudes, and intentions, which are key drivers of vaccine uptake [41].

Perceptions of potential side effects of the HPV vaccine were associated with vaccine hesitancy. In Ethiopia, a survey of 423 adolescent girls showed that concerns about vaccine safety were a major barrier to girls’ adherence to vaccination [42].

The HPV vaccination schedule typically requires one or two doses administered at specific intervals. For an adolescent girl who is unaware of the exact number of doses or the timing between injections, the process may appear complicated, as it involves multiple visits and careful follow-up [43]. It becomes uncertain because the young person may wonder if a missed or delayed dose reduces effectiveness or if she will complete the entire schedule correctly. This sense of difficulty and uncertainty heightens doubt and anxiety, ultimately fostering vaccination hesitancy. In our study, a lack of knowledge about the number of doses to administer was associated with vaccination hesitancy. Reminder and follow-up systems—such as SMS notifications, electronic reminders, and school-based follow-ups—have been found to increase adherence to multi-dose vaccination schedules and reduce drop-offs between doses [44].

Fear of vaccination is a major driver of vaccine hesitancy and is often rooted in concerns about injection pain, possible side effects, or rumors surrounding vaccine safety [45]. Among adolescent girls, this fear may be heightened by worries about pain, local reactions such as redness or swelling, or adverse effects that are perceived rather than commonly observed. From a psychological perspective, individuals may let the anticipation of discomfort or negative effects outweigh the perceived benefits of vaccination, ultimately leading them to hesitate [45]. In our study, fear of vaccination emerged as a significant factor contributing to HPV vaccine hesitancy. Adolescent-friendly service delivery, which includes measures to lessen pain during vaccination and supportive counseling approaches, can improve vaccination experiences and reduce fear, hence increasing acceptability.

Similarly, fear of anticipatory regret is a psychological determinant of hesitancy regarding HPV vaccination. Our study confirmed this finding. Many adolescent girls worry that they might regret getting vaccinated if side effects occur, and that fear heightens their uncertainty, causing them to delay their decision. This mechanism of anticipatory regret, well-documented in health psychology, directly influences the propensity to accept or refuse a vaccine [46]. In the case of HPV in America, a survey conducted on a sample of 1,484 participants found fear of anticipatory regret to be a risk factor associated with refusing or reporting vaccination, despite recognition of its preventive benefits [47]. Behavioral communication strategies that frame vaccination benefits positively and address cognitive biases such as anticipatory regret—emphasizing protection and long-term health outcomes—have been shown to influence decision-making favorably and increase vaccine acceptance [48].

## Limitation

Some limitations must be considered. The cross-sectional nature of the study does not allow for establishing causality, as exposure and outcome were assessed simultaneously. Potential selection bias may have occurred if girls aged 11–14 years who were in school differed from those who were not. Furthermore, recall bias may have affected self-reported data regarding vaccination history and healthcare experiences. Finally, social desirability bias cannot be ruled out due to the face-to-face data collection.

## Conclusion

The percentage of HPV vaccination hesitancy among young girls remains high. Several factors contribute to this, including the influence of unvaccinated peers, parental illiteracy, limited recommendations from health professionals, prevailing sociocultural beliefs, and inadequate knowledge about the vaccine. Our findings highlight the importance of implementing targeted educational initiatives that enhance young people’s understanding of the vaccine’s efficacy and safety, while also strengthening their sense of personal and community responsibility for health. Furthermore, it is important to raise awareness among health professionals, encouraging them to routinely recommend vaccination to parents and to integrate vaccination sessions into school health programs.

## Abbreviations

AOR: Adjusted odds ratio
CI: Confidence interval
HPV: human papillomavirus
OR: Odds ratio
WHO: World health organization
